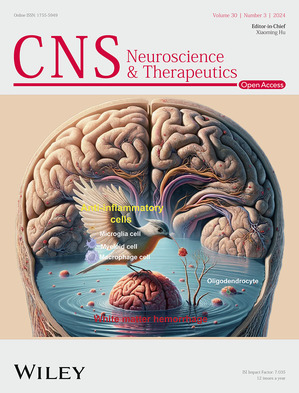# Front Cover

**DOI:** 10.1111/cns.14734

**Published:** 2024-05-17

## Abstract

The cover image is based on the Original Article *Unraveling the complex pathophysiology of white matter hemorrhage in intracerebral stroke: A single‐cell RNA sequencing approach* by Lisha Ye et al., https://doi.org/10.1111/cns.14652.